# MRI criterion for prediction of involvement of circumferential resection margin in rectal cancer

**DOI:** 10.4103/0971-3026.76065

**Published:** 2011

**Authors:** Mudit Gupta, Rashmi Gupta

**Affiliations:** The Ottawa Hospital, Box 232, General Campus-Room 1466e, 501 Smyth Road, Ottawa, Ontario K1H8L6, Canada E-mail: drmuditgupta@yahoo.co.in

Dear Sir,

We read the article “MRI in T staging of rectal cancer: How effective is it?" by Mulla *et al*.,[1] published recently in this journal. We agree with the authors that MRI is moderately accurate in T staging for rectal cancer. As pointed out by the authors, the status of the circumferential resection margin (CRM) is very important as this will decide whether a patient needs neoadjuvant chemo radiotherapy before surgery. Involvement of the CRM is an independent predictor of increased chance of recurrence after surgery. Currently, MRI is the modality of choice to evaluate the status of the CRM.

However, it would be interesting to know the criterion used by the authors for predicting the involvement of the CRM. Beets *et al*.[2] have shown that on MRI, a distance of 6 mm will predict a tumor distance of at least 2 mm on histology with 97% confidence and a crucial distance of 1 mm can be predicted by a distance of 5 mm on MRI with high confidence. Other authors[3] have also confirmed the same fact in their studies, and currently on MRI, 5 mm is the cutoff used for prediction of involvement of the CRM in our institution. Any tumor within 5 mm of the CRM will have a high chance of involvement of the CRM.
